# Neuroprotective effect of 3,3’-Diindolylmethane against perinatal asphyxia involves inhibition of the AhR and NMDA signaling and hypermethylation of specific genes

**DOI:** 10.1007/s10495-020-01631-3

**Published:** 2020-08-20

**Authors:** J Rzemieniec, E Bratek, A Wnuk, K Przepiórska, E Salińska, M. Kajta

**Affiliations:** 1 Laboratory of Molecular Neuroendocrinology, Department of Experimental Neuroendocrinology, Maj Institute of Pharmacology, Polish Academy of Sciences, 12 Smetna Street, 31-343 Krakow, Poland; 2 Department of Neurochemistry, Mossakowski Medical Research Centre, Polish Academy of Sciences, 5 Pawinskiego Street, 02-106 Warsaw, Poland

**Keywords:** Hypoxia/ischemia, Perinatal asphyxia, 3,3’-diindolylmethane, Neuroprotection, AhR, NMDA

## Abstract

Each year, 1 million children die due to perinatal asphyxia; however, there are no effective drugs to protect the neonatal brain against hypoxic/ischemic damage. In this study, we demonstrated for the first time the neuroprotective capacity of 3,3’-diindolylmethane (DIM) in an in vivo model of rat perinatal asphyxia, which has translational value and corresponds to hypoxic/ischemic episodes in human newborns. Posttreatment with DIM restored the weight of the ipsilateral hemisphere and normalized cell number in the brain structures of rats exposed to perinatal asphyxia. DIM also downregulated the mRNA expression of HIF1A-regulated *Bnip3* and *Hif1a* which is a hypoxic marker, and the expression of miR-181b which is an indicator of perinatal asphyxia. In addition, DIM inhibited apoptosis and oxidative stress accompanying perinatal asphyxia through: downregulation of FAS, CASP-3, CAPN1, GPx3 and SOD-1, attenuation of caspase-9 activity, and upregulation of anti-apoptotic *Bcl2* mRNA. The protective effects of DIM were accompanied by the inhibition of the AhR and NMDA signaling pathways, as indicated by the reduced expression levels of AhR, ARNT, CYP1A1, GluN1 and GluN2B, which was correlated with enhanced global DNA methylation and the methylation of the *Ahr* and *Grin2b* genes. Because our study provided evidence that in rat brain undergoing perinatal asphyxia, DIM predominantly targets AhR and NMDA, we postulate that compounds that possess the ability to inhibit their signaling are promising therapeutic tools to prevent stroke.

## Introduction

Perinatal asphyxia affects 2–4 newborns per 1000 births. Each year, 1 million children die due to hypoxia [1]. Oxygen deprivation in the perinatal period can lead to permanent brain damage and result in the onset of hypoxic-ischemic encephalopathy (HIE). HIE is the most serious consequence of perinatal asphyxia and has a wide spectrum of symptoms, such as cerebral palsy, convulsions, mental and motor impairment, and speech, hearing or visual disturbances. Currently, the gold standard for reducing brain damage induced by asphyxia is oxygen therapy and moderate hypothermia [2]. However, there is still a lack of effective and safe compounds that protect children’s brains from hypoxia/ischemia-induced damage.

A promising neuroprotective agent is 3,3’-diindolylmethane (DIM). DIM is a selective aryl hydrocarbon receptor modulator (SAhRM) found in cruciferous vegetables such as broccoli, Brussels sprouts, cabbage and kale. The SAhRM properties of DIM make this compound very attractive from a pharmacological point of view; since DIM acts as an AhR agonist or antagonist in a tissue-specific manner, it may exert beneficial effects on the central nervous system and in peripheral tissues. Recent data have shown that experimental stroke is followed by an increase in AhR/ARNT expression levels in neurons in vitro and in vivo [3, 4]. In experimental studies, DIM administered orally to mice at a dose of 250 mg/kg reached a concentration of 5–36.5 µM in the brain within 6 h of its administration [5]. Furthermore, our previous study demonstrated that DIM strongly protected mouse primary hippocampal cells against hypoxia/ischemia [6, 7] that inspired us to test the neuroprotective capacity of the compound in an in vivo model of rat perinatal asphyxia.

It is well known that excitotoxicity is a key mechanism of cell death during hypoxia/ischemia. Activation of N-methyl-D-aspartate receptors (NMDARs) followed by oxidative stress and mitochondrial failure leads to apoptosis and/or necrosis of brain cells [8]. During the neonatal period of life, NMDA receptor-coupled channels have a higher probability of aperture and conductance than adult channels. Thus, increased expression of the GluN1 and GluN2B subunits is responsible for greater excitability. Opened NMDA receptor-coupled channels allow calcium ions to enter the intracellular compartment and activate calcium-dependent enzymes, i.e., caspases and calpains; this activation leads to apoptotic and necrotic cell death [9, 10]. Interestingly, knockdown of AhR attenuates NMDA-mediated excitotoxicity in cortical neurons, which points to probable crosstalk between these two receptors [11]. However, there are no data on the neuroprotective effectiveness of DIM during perinatal asphyxia. Knowledge about the roles of the AhR and NMDA signaling pathways in the action of DIM in the rat brain during asphyxia is negligible.

We recently showed that DIM protects neurons against hypoxia/ischemia via inhibition of apoptosis [6, 7]. However, there are no data on the effects of DIM on the apoptosis-signaling pathway in a model of perinatal asphyxia. It has been shown that newborns suffering from perinatal asphyxia have higher plasma levels of glutathione peroxidase and superoxide dismutase compared to control subjects [12]. Glutathione peroxidase (GPx) and superoxide dismutase (SOD) are anti-oxidant enzymes that protect biological structures from free oxygen radical-mediated injury. Nevertheless, there are no data concerning the effect of DIM on the expression of GPx and SOD in the brains of rats subjected to perinatal asphyxia.

There is an increasing body of evidence indicating the influence of perinatal asphyxia on epigenetic modifications, including DNA methylation and microRNA (miRNA) expression, which in turn regulate the expression of target genes. Many efforts have been made to find a reliable biomarker of ischemic stroke. It has been demonstrated that the expression of miRNAs such as miR-124, miR-181b, miR-384 and miR-223 is variable both in animals subjected to ischemia and in stroke patients [13, 14]. However, there is no information on the functional significance of miRNAs in the brains of rats subjected to perinatal asphyxia and treated with DIM. In addition, it is not clear whether DIM causes hypo- or hypermethylation of DNA.

Therefore, the aim of our study was to assess the neuroprotective capacity of 3,3′-diindolylmethane in a rat model of perinatal asphyxia. Particular attention was paid to the influence of DIM on the AhR and NMDA signaling, the epigenetic status of asphyxic brain as well as on apoptosis and oxidative stress.

## Materials and methods

### Materials

3,3’-diindolylmethane, and corn oil, RIPA buffer, protease inhibitor cocktail for mammalian tissues and cresyl violet were purchased from Sigma-Aldrich (St. Louis, MO, USA). A cDNA reverse transcription kit, TaqMan Gene Expression Master Mix and TaqMan probes for specific genes such as *Ahr, Arnt, Cyp1a1, Hif1a, Grin2b, Gpx3, Sod1, Ahrr and Bnip3* were obtained from Life Technologies Applied Biosystems (Carlsbad, CA, USA). High Capacity cDNA-Reverse Transcription Kit was obtained from Thermo Fisher Scientific (Massachusetts, USA). BM Chemiluminescence Western Blotting Substrate (POD) and an mRNA isolation kit were purchased from Roche Diagnostics GmbH (Mannheim, Germany). Laemmli sample buffer (2x), Bradford reagent, 7.5% and 10% Mini-PROTEAN TGX precast gels were obtained from Bio-Rad Laboratories (Hercules, CA, USA). Immobilon-P membranes were obtained from Merck Millipore (Burlington, MA, USA). A mouse monoclonal anti-β-actin antibody (sc-47,778), mouse monoclonal anti-NMDA ε2 antibody (sc-365,597), goat polyclonal anti-NMDA ε2 antibody (sc-1469), rabbit polyclonal anti-AhR antibody (sc-5579), goat polyclonal CYP1A1 antibody (sc-9828), rabbit polyclonal anti-BAX antibody (sc-493), and mouse monoclonal anti-BCL2 antibody (sc-7382) were purchased from Santa Cruz Biotechnology, Inc. (Santa Cruz, CA, USA). An anti-rabbit cleaved CASPASE-3 antibody (9661) was obtained from Cell Signaling Technology (Danvers, MA, USA). ELISA kits for AhR, ARNT, and HIF1A were purchased from Wuhan EIAab (Wuhan, China), and an ELISA kit for CAPN1 was purchased from Qayee Bio-Technology Co., Ltd. (Shanghai, China). A Caspase 9 Assay Kit and a goat polyclonal anti-GPX-3 antibody were obtained from Abcam (Cambridge, United Kingdom). An EpiTect MethyLight PCR Kit, miRCURY LNA RT Kit, miRCURY LNA SYBR Green PCR Kit, RNeasy and miRNeasy Mini Kit and specific primers for *miR-124-3p*, *miR-223-3p*, *miR-181b-5p*, *miR 384-5p* and *UniSp6* were obtained from Qiagen (Valencia, CA, USA). An EZ DNA Methylation-Gold™ Kit and Quick-gDNA™ MicroPrep Kit were obtained from Zymo Research (Irvine, CA, USA).

### Induction of perinatal asphyxia (hypoxia/ischemia)

Neonatal cerebral hypoxia/ischemia (HI) was induced according to the methods described by Rice et al. [15]. Briefly, 7-day-old Wistar rat pups of both sexes (weight, 12–18 g) were anesthetized with isoflurane (4% for induction and 1.5–2.0% for maintenance) in a mixture of nitrous oxide and oxygen (0.6:1). The left common carotid artery was exposed and cut between double ligatures of silk sutures or was only exposed (sham control). Before closing, the wound was treated with local anesthetic (lignocainum). After 60 min of recovery, the animals were placed in a humidified chamber (35 °C) and exposed to a hypoxic gas mixture (7.5% oxygen in nitrogen) for 75 min as previously described [16]. After hypoxic treatment, the animals were returned to their cages and housed with their mother at room temperature (22 °C) with a 12:12-h light-dark cycle and ample food and water. The condition of the animals, which stayed in the experiment for fourteen days, was checked twice a day.

### Drug application

Seven-day-old Wistar rat pups were injected intraperitoneally (i.p.) with 3,3’-diindolylmethane 30 min, 24 h, 48 and 72 h after HI at a dose of 0.1, 10 or 100 mg/kg of body weight. These doses were determined based on the previously published findings of [17–19]. Sham-operated and HI control rats were injected with corn oil.

### Evaluation of brain damage

Fourteen days after HI, the rat brains were removed, and both cerebral hemispheres were weighed. Brain damage was reflected by a decrease in the wet weight of the ipsilateral (left) ischemic hemisphere, which was expressed as the percentage of the wet weight of the contralateral (right) control hemisphere, as previously described [20].

### qPCR analysis of the mRNA levels of genes encoding *Ahr*, *Arnt*, *Cyp1a1*, *Ahrr*, *Hif1a*, *Bnip3*, *Bax*, *Bcl2*, *Grin2b*, *Gpx3* and *Sod1*

Total RNA (approximately 1 µg of RNA per sample) was isolated from brain tissues 3 days after hypoxia/ischemia using the RNeasy Mini Kit (Qiagen, Valencia, CA). The quantity of RNA was spectrophotometrically determined at 260 nm and 260/280 nm (ND/1000 UV/Vis; Tecan NanoDrop, USA). cDNA was synthesized using the High Capacity cDNA-Reverse Transcription Kit (Thermo Fisher Scientific, USA). The reverse transcription reaction and quantitative polymerase chain reaction (qPCR) were run on the CFX96 Real-Time System (Bio-Rad, Hercules, CA, USA) as previously described [21]. Amplification was performed in a total volume of 20 µl containing 10 µl of TaqMan Gene Expression Master Mix and 1.0 µl of reverse transcription product as the PCR template. A standard qPCR procedure was utilized: 2 min at 50 °C and 10 min at 95 °C followed by 40 cycles of 15 s at 95 °C and 1 min at 60 °C. The threshold value (Ct) for each sample was set during the exponential phase, and the delta Ct method was used for data analysis. To evaluate reference gene expression, the RefFinder web-based comprehensive tool was used [22]. For our study, NormFinder, BestKeeper and delta Ct recommended *Hprt* as the most stable reference gene.

### qPCR analysis of specific miRNAs

miRNA (approximately 300 ng of RNA per sample) was isolated from the brains of 10-day-old rat pups subjected to HI and/or treated with 3,3’-diindolylmethane (10 mg/kg) using the miRNeasy Mini Kit (Qiagen, Valencia, CA). The quantity of RNA was spectrophotometrically determined at 260 nm and 260/280 nm (ND/1000 UV/Vis; Tecan NanoDrop, USA). First-strand cDNA synthesis (optimized for 20 ng of RNA) was performed using the miRCURY LNA RT Kit (Qiagen, Valencia, CA) according to the manufacturer’s protocol. Quantitative polymerase chain reaction (qPCR) was conducted with the miRCURY SYBR Green PCR Kit and miRCURY LNA miRNA PCR custom-made assays (Qiagen, Valencia, CA). The reverse transcription reaction and qPCR were run on the CFX96 Real-Time System (Bio-Rad, Hercules, CA, USA) as previously described [23]. The threshold value (Ct) for each sample was set during the exponential phase, and the delta-delta Ct method was used for data analysis. The *U6* coding gene was employed as a reference gene based on the recommendations of the statistical tools of BestKeeper, delta-Cq, and NormFinder.

### Histochemistry

One week after perinatal asphyxia, animals from each experimental group were sacrificed for cresyl violet staining (n = 3–5). The animals were anesthetized by i.p. ketamine (90 mg/kg body weight) and xylazine (10 mg/kg body weight) injections and subjected to intracranial perfusion with 4% neutralized formalin for fixation (Sigma-Aldrich, St. Louis, MO, USA). The brains were removed, immersed in 4% formalin for 4 h, transferred to absolute ethanol and embedded in paraffin. Ten-micron cross-sections of the dorsal part of the hippocampus (between 2.2 and 3.5 mm posterior to bregma) were used to evaluate neuronal cell damage in the cerebral cortex and hippocampus. The sections were stained with cresyl violet (Sigma, St. Louis, MO, USA), as previously described [24]. For each animal, at least five sections of the cerebral cortex and the central part of the CA1 region of both hippocampi were analyzed for neuronal density (3–5 animals per group). The number of neurons was counted using the AxioVision imaging program (Carl Zeiss, Aalen, Germany). The mean number of neurons stained with cresyl violet was expressed as the percentage of the mean number of neurons in sham-operated rats.

### Western blot analyses

Three and seven days after perinatal asphyxia, brain tissues were isolated and homogenized in 10 mM PBS pH 7.4 containing 10 mM EGTA, 10 mM EDTA, 100 mM NaCl, 0.1 mM PMSF and protease inhibitor cocktail (Sigma-Aldrich, St. Louis, MO, USA). The protein concentrations in the supernatants were determined using Bradford reagent with bovine serum albumin (BSA) as the standard. Samples containing 40 µg of total protein were reconstituted in the appropriate amount of Laemmli sample buffer, denatured (95 °C, 5 min), and separated on 7.5 and 10% SDS-polyacrylamide gels using a Bio-Rad Mini-Protean 3 system as previously described [25]. After electrophoresis, the proteins were transferred onto PVDF membranes using a Bio-Rad Mini Trans-Blot apparatus. Afterwards, the nonspecific binding sites were blocked with 5% nonfat dry milk and 0.2% Tween-20 in 0.02 m TBS (Tris-buffered saline) for 1.5 h with shaking. Then, the membranes were incubated overnight (at 4 °C) with one of the following primary antibodies in 2.5% nonfat dry milk and 0.1% TBS/Tween: mouse monoclonal anti-β-actin antibody (diluted 1:3000), rabbit polyclonal anti-AhR antibody (diluted 1:100), rabbit polyclonal anti-ARNT antibody (diluted 1:100), goat polyclonal anti-CYP1A1 (diluted 1:150), rabbit polyclonal anti-FAS antibody (diluted 1:100), mouse monoclonal anti-BAX antibody (diluted 1:100), rabbit polyclonal anti-cleaved Caspase-3 antibody (diluted 1:1000), goat polyclonal anti-NR1 antibody (diluted 1:1000), goat polyclonal anti-NR2B antibody (diluted 1:1000), goat polyclonal anti-GPx-3 antibody (diluted 1:400), mouse monoclonal anti-SOD-1 antibody (diluted 1:1000). Subsequently, the membranes were washed 5 times with 4% nonfat milk with TBS and 0.2% Tween 20 and incubated for 1 h with horseradish peroxidase-conjugated secondary antibodies (goat anti-rabbit IgG or goat anti-mouse IgG) diluted 1:1000 or/and 1:3000 in 0.25% nonfat milk with TBS/Tween. The images were developed using BM Chemiluminescence Blotting Substrate (Roche Diagnostics GmBH, Mannheim, Germany) and visualized using a Luminescent Image Analyzer Fuji-Las 4000 (Fuji, Japan). The immunoreactive bands were quantified using an image analyzer (ScienceLab, MultiGauge V3.0).

### ELISAs for AhR, ARNT, HIF1A, and CALPAIN-1

The levels of AhR, ARNT, HIF1A, and CALPAIN-1 (CAPN1) in brains isolated 3 and 7 days after perinatal asphyxia were determined via ELISA. Detection of these proteins was achieved using commercially available quantitative sandwich enzyme immunoassay kits as previously described [26]. The absorbance was measured at 450 nm and was proportional to the amount of AhR, ARNT, HIF1A, and CAPN1. The protein concentration of each sample was determined using the Bradford reagent (Bio-Rad Protein Assay, Hercules, CA, USA).

### Measurement of caspase-9 activity

Tissues from the left hemisphere were homogenized separately in 50 mM potassium orthophosphate, pH 7.0, containing 1 mM EDTA. The homogenates were incubated in RIPA buffer for 1 h at 4 °C, and then the samples were centrifuged for 10 min at 10,000 × g at 4 °C. After centrifugation, the lysates were transferred to a new cooled 1.5-ml polyethylene tube. The protein concentration (Bradford method) was measured, and the samples were frozen at − 80 °C for further determination. Caspase-9 activity was determined by an immunoenzymatic method (ELISA (enzyme-linked immunosorbent assay)) using a Caspase-9 Assay Kit (fluorometric, Abcam) according to the manufacturer’s instructions.

### Measurement of global DNA methylation

An Imprint Methylated DNA Quantification Kit was used to determine the methylation status of the DNA in brain tissues 3 days after hypoxia/ischemia. Methylated DNA was detected using capture and detection antibodies and quantified calorimetrically as previously described [27]. Purified DNA in the amount of 50 ng per well was added to a 96-well plate. After DNA binding and incubation with the capture antibody, a developing solution was added to monitor the reactions for color changes. The absorbance was read at 450 nm, and the relative global methylation levels were calculated. For each sample, the DNA quantity was determined spectrophotometrically (ND/1000 UV/Vis; Thermo Fisher NanoDrop, USA).

### Measurement of DNA methylation of the *Ahr* and *Grin2b* genes

Genomic DNA was extracted from brain tissues 3 days after HI using the Quick-gDNA™ MicroPrep (Zymo Research, Irvine, CA) according to the manufacturer’s protocol. The quantity of DNA was spectrophotometrically determined at 260 nm and 260/280 nm (ND/1000 UV/Vis; Thermo Fisher NanoDrop, USA). Sodium bisulfite conversion of genomic DNA was performed with the EZ DNA Methylation-Gold™ Kit (Zymo Research, Irvine, CA). The bisulfite-converted samples were eluted in a 10 µl volume and stored at − 80 °C until use. We performed quantitative real-time polymerase chain reaction (MethyLight) using an EpiTect MethyLight PCR Kit (Qiagen, Valencia, CA). Sets of TaqMan probes designed specifically for bisulfite converted DNA sequences, a set of fully methylated and fully unmethylated probes for the *Ahr* and *Grin2b* promoters and an internal reference set for the *Hprt* gene to control for input DNA, were used. The relative level of methylation was determined by the 2^−ΔΔCt^ method, according to the following formula: ΔΔCt = methylated signal (Ct target gene − Ct *Hprt*) − unmethylated signal (Ct target gene − Ct *Hprt*) [28].

### Ethics approval and consent for participation

All experiments were approved by the 2nd Local Ethical Committee based in Warsaw, Poland and were performed in accordance with Polish governmental regulations (Dz.U.97.111.724), with the European Community Council Directive of 24 November 1986 (86/609/EEC) and Directive 2010/63/EU. All surgeries were performed under isoflurane anesthesia, and all efforts were made to minimize animal suffering and the number of animals used.

### Data analysis

The data were statistically analyzed as follows: fluorescence units per 1 µg of RNA (qPCR), pg of specific protein per µg of total protein (ELISA), mean optical density per 40 µg of protein (Western blot), ng per gram of tissue, and ng of methylated DNA per 50 ng of DNA sample (global DNA methylation). The number of animals per group was 6. The results are expressed as means ± SEMs. To compare the effects of perinatal asphyxia and DIM in the rat pups, the global DNA methylation and western blot analysis results are presented as the percentage of the control. The data were analyzed by one-way ANOVA. The analyses were preceded by Levene’s test of homogeneity of variance and used to determine the overall significance. The differences between the control and experimental groups were assessed with a post hoc Neuman-Keuls test. Significant differences are indicated as follows: *p < 0.05, **p < 0.01, and ***p < 0.001 (versus the sham rats) and #p < 0.05, ##p < 0.01, and ###p < 0.001 (versus the animals exposed to perinatal asphyxia). Statistical analysis of the brain damage data was performed via paired t test. Statistical analysis of caspase-9 activity was performed by one-way ANOVA, with further analysis involving a post hoc least significance test for significant differences between groups was performed (GraphPad Prism, version 5.01; GraphPad Software Inc., La Jolla, California, USA). Differences were considered significant when p values of less than 0.05 were found.

## Results

### DIM reduces hypoxia/ischemia-induced brain damage

**Fig. 1:**
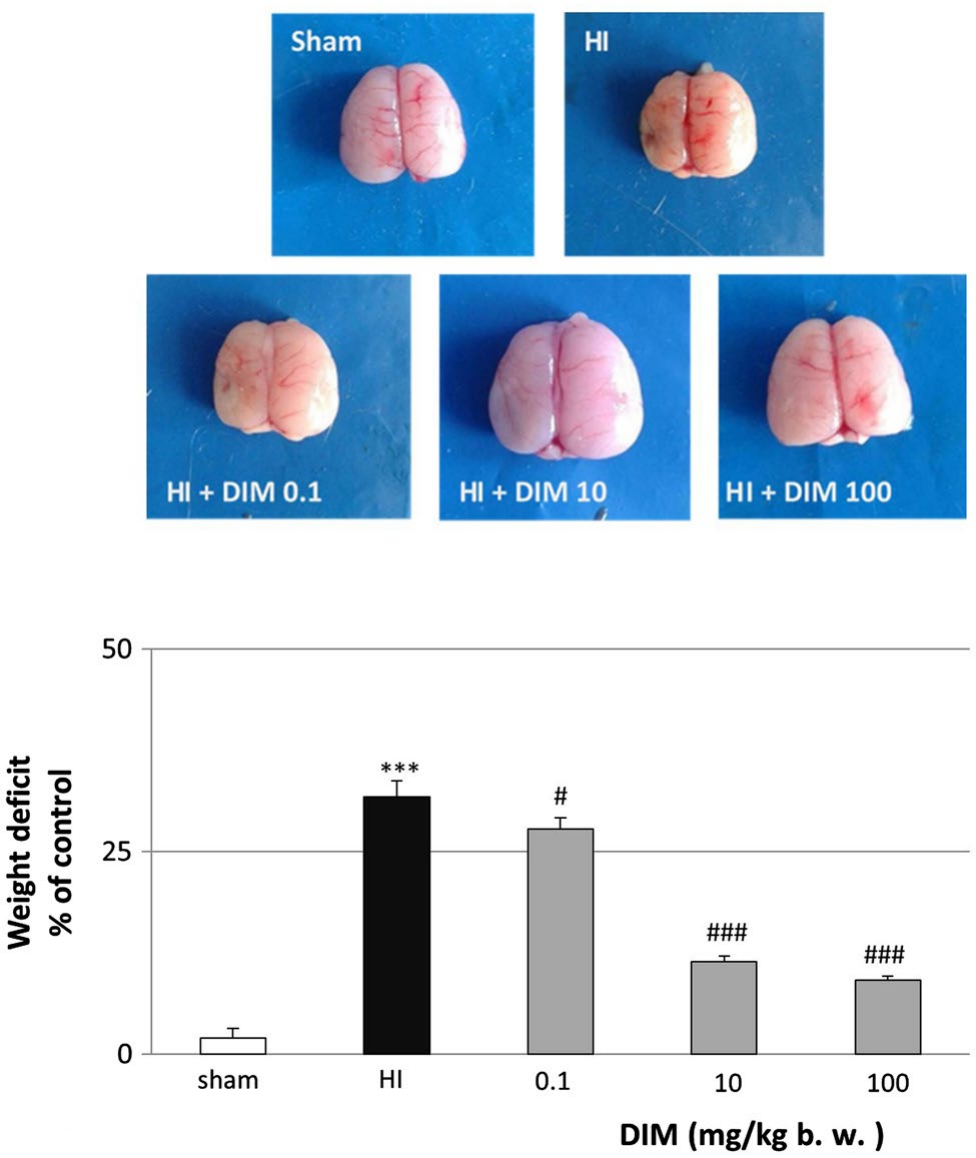
DIM (0.1–100 mg/kg) inhibits hypoxia/ischemia-induced weight deficit in the ipsilateral hemisphere. Seven-day-old rat pups were injected intraperitoneally (i.p.) with DIM 0.5 h, 24 h, 48 and 72 h after hypoxia/ischemia (HI) at a dose of 0.1, 10 or 100 mg/kg of body weight. Sham-operated and HI control rats were injected with corn oil. The weight deficit is expressed as the percentage of the weight of the contralateral (left) hemisphere. The results are presented as the mean values ± SEMs, n = 3–5. ***p < 0.001 versus sham animals, #p < 0.05 and ###p < 0.001 versus hypoxia/ischemia-treated animals

In rat pups subjected to hypoxia/ischemia (HI), damage to the ipsilateral hemisphere was assessed 14 days after the insult. In untreated animals, the hypoxia/ischemia-induced weight deficit reached 31.7%. Prolonged treatment with 3,3’-diindolylmethane (0.1–100 mg/kg) (injected intraperitoneally 30 min after insult and then for 3 days at 24-h intervals) resulted in a reduction in the weight deficit ranging from 9.2 to 27.8% depending on the dose (Fig. 1). In sham-operated animals, there was no difference between the masses of the left and right hemispheres (result not shown).

For further experimentation, we chose a DIM dose of 10 mg/kg, which induced a strong neuroprotective effect after hypoxia/ischemia.

### DIM prevents hypoxia/ischemia-induced neuronal cell loss in the hippocampal CA1 and cerebral cortex

**Fig. 2:**
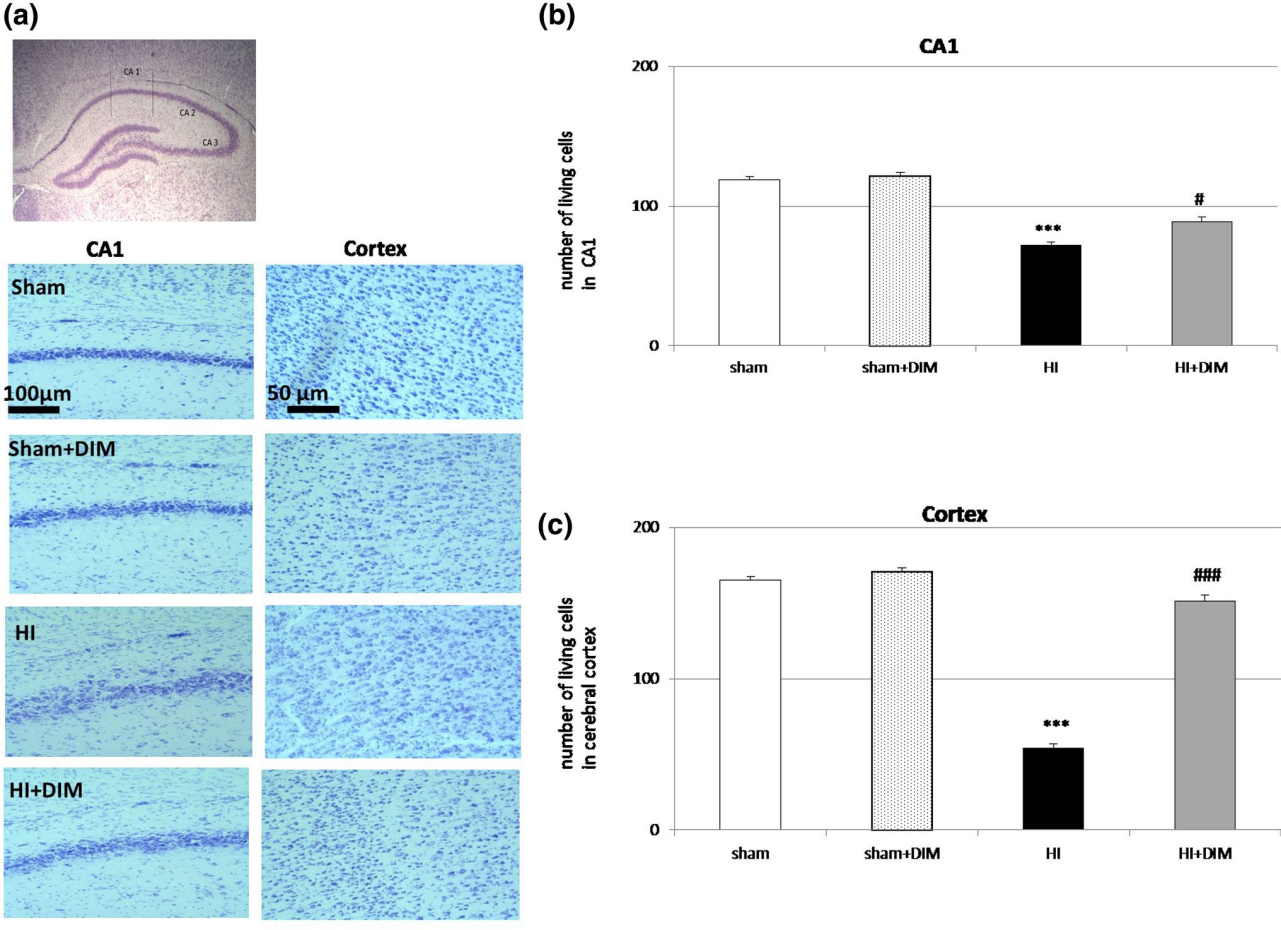
DIM prevents hypoxia/ischemia-induced neuronal cell loss in the hippocampal CA1 region and cerebral cortex. Seven-day-old rat pups were injected intraperitoneally (i.p.) with DIM 0.5 h, 24 h, 48 and 72 h after hypoxia/ischemia (HI) at a dose of 10 mg/kg of body weight. Sham-operated and HI control rats were injected with corn oil. Histochemical evaluation of cerebral damage was performed 7 days after HI. Analyzed area: central part of the CA1 region marked with vertical lines (100 µm length) and cortex (250 µm x 250 µm). The microphotographs show the ipsilateral hemisphere. ***p < 0.001 versus sham animals, #p < 0.05, ###p < 0.001 versus hypoxia/ischemia-treated animals

Cresyl violet staining showed hypoxia/ischemia-evoked damage and disorganization of neurons in the CA1 region of the hippocampus and cerebral cortex. The number of neurons in the analyzed asphyxic areas of the CA1 region (marked with vertical lines 100 µm length) and cortex (250 µm x 250 µm) was reduced to 71 ± 4 and 54 ± 5 compared to 118 ± 3 and 170 ± 8 in the controls, respectively. The intraperitoneal injection of 10 mg/kg DIM significantly increased the number of living cells to 88.5 ± 5 and 151 ± 4 in the CA1 region and cortex, respectively. The administration of DIM (10 mg/kg) to sham animals did not evoke any changes in the hippocampal CA1 region and cortex organization (Fig. 2).

### DIM inhibits hypoxia/ischemia-induced changes in the expression of mRNAs and/or proteins belonging to AhR signaling pathways three and seven days after the insult

#### DIM inhibits the hypoxia/ischemia-induced changes in mRNA and/or protein expression levels of *Ahr*/AhR, *Arnt*/ARNT, *Cyp1a1*/CYP1A1 and *Ahrr* observed three days after the insult

**Fig. 3:**
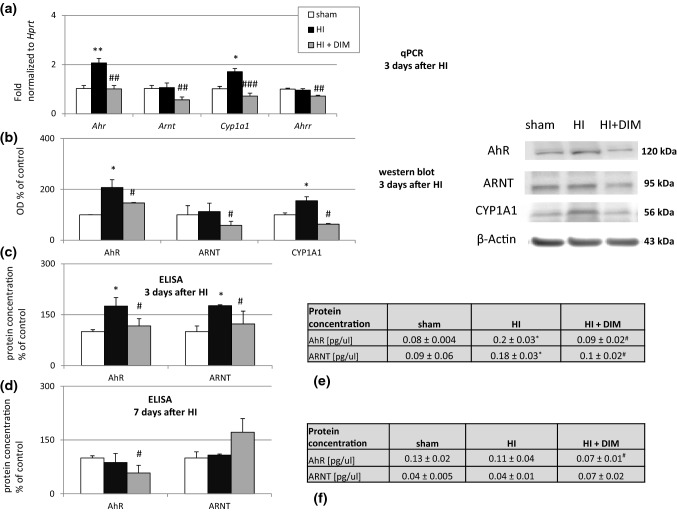
DIM inhibits hypoxia/ischemia-induced changes in the mRNA (**a**) and/or proteins (**b–f**) expression related to AhR signaling pathways three and seven days after insult. Seven-day-old rat pups were injected intraperitoneally (i.p.) with DIM 0.5 h, 24 h, 48 and 72 h after hypoxia/ischemia (HI) at a dose of 10 mg/kg of body weight. Sham-operated and HI control rats were injected with corn oil. The results are presented as the fold change and were normalized to *Hprt* or are presented as a percentage of the control. Each bar represents the mean ± SEM. *p < 0.05, **p < 0.01 versus sham animals, #p < 0.05, ##p < 0.01, and ###p < 0.001 versus hypoxia/ischemia-treated animals

In our model, hypoxia/ischemia caused increases in the mRNA expression of *Ahr* and *Cyp1a1* ranging from 1.7 to 2-fold but did not evoke changes in the mRNA expression of *Arnt* and *Ahrr.* The administration of DIM (10 mg/kg) decreased the expression of *Ahr*, *Arnt, Cyp1a1* and *Ahrr* from 0.5-fold to 1-fold relative to the control level (Fig. 3a).

Western blot analyses revealed that hypoxia/ischemia increased the protein levels of AhR and CYP1A1 in the rat brains to 207% and 155% of control levels, respectively. Under hypoxic/ischemic conditions, DIM significantly reduced the protein expression levels of AhR, ARNT and CYP1A1 to 146%, 58%, and 63% of control levels, respectively (Fig. 3b).

The results obtained from ELISA showed that the levels of AhR and ARNT increased in the rat brains after hypoxia/ischemia to approximately 175% of control levels. Under hypoxic/ischemic conditions, DIM evoked a decrease in the AhR and ARNT protein concentration to approximately 120% (Fig. 3c). Figure 3e presents the concentrations of selected proteins in pg/µl.

#### DIM decreases the hypoxia/ischemia-induced protein concentration of AhR seven days after the insult

We did not observe any changes in the AhR or ARNT protein concentration 7 days after hypoxia/ischemia. However, at this time, DIM decreased the AhR protein concentration to 58% of the control level (Fig. 3d). Figure 3f presents the concentrations of selected proteins in pg/µl.

### DIM inhibits hypoxia/ischemia-induced changes in expression of mRNAs and proteins belonging to the NMDA signaling pathway three and seven days after the insult

#### DIM decreases the mRNA and protein expression levels of NMDA subunits three days after the insult

**Fig. 4:**
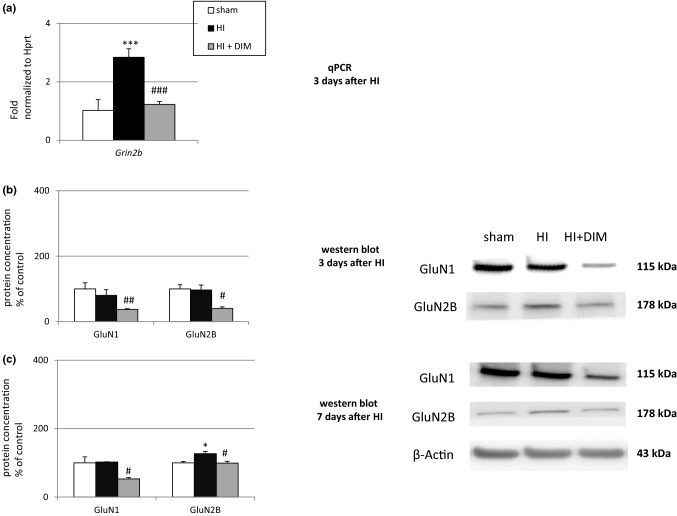
DIM inhibits hypoxia/ischemia-induced changes in mRNA (**a**) and proteins expression (**b**, **c**) related to the NMDA signaling pathway three and seven days after insult. Seven-day-old rat pups were injected intraperitoneally (i.p.) with DIM 0.5 h, 24 h, 48 and 72 h after hypoxia/ischemia (HI) at a dose of 10 mg/kg of body weight. Sham-operated and HI control rats were injected with corn oil. The relative protein levels are presented as a percentage of the control. Each bar represents the mean ± SEM. *p < 0.05, and ***p < 0.001 versus sham animals, #p < 0.05, ##p < 0.01, and ###p < 0.001 versus hypoxia/ischemia-treated animals

In our experimental model of perinatal asphyxia, hypoxia/ischemia caused an almost 3-fold increase in *Grin2b* mRNA expression measured 3 days after insult. Administration of DIM (10 mg/kg) significantly decreased the expression of *Grin2b* mRNA to 1.2-fold relative to the control level (Fig. 4a).

Western blot analyses revealed that hypoxia/ischemia did not change the protein level of GluN1 and GluN2B three days after insult. However, treatment with DIM significantly reduced the protein expression levels of both subunits to approximately 40% of control (Fig. 4b).

#### DIM decreases the hypoxia/ischemia-induced protein level of GluN1 and GluN2B seven days after the insult

Seven days after perinatal asphyxia, the protein level of GluN2B increased by approximately 30% compared to control values, whereas the level of GluN1 did not change. DIM reduced the expression of GluN1 and GluN2B to 53% and 99% of the control level, respectively (Fig. 4c).

### DIM inhibits the hypoxia/ischemia-induced increase in the expression of HIF1A and CALPAIN-1 three and seven days after the insult

#### DIM inhibits the hypoxia/ischemia-induced mRNA expression of *Hif1a* and *Bnip3* but it did not change the protein levels of HIF1A or CALPAIN-1 three days after the insult

**Fig. 5:**
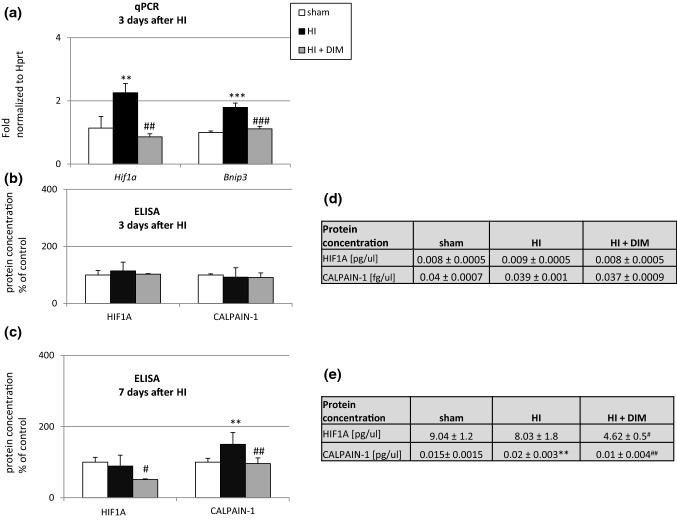
DIM inhibits the mRNA (**a**) and/or protein (**b**) expression of HIF1A, BNIP3 and CALPAIN-1 three and seven days after insult. Seven-day-old rat pups were injected intraperitoneally (i.p.) with DIM 0.5 h, 24 h, 48 and 72 h after hypoxia/ischemia (HI) at a dose of 10 mg/kg of body weight. Sham-operated and HI control rats were injected with corn oil. Each bar represents the mean ± SEM. The number of replicates ranged from 6 to 8 (qPCR and ELISA). **p < 0.01 and ***p < 0.001 versus sham animals, #p < 0.05, ##p < 0.01, and ###p < 0.001 versus hypoxia/ischemia-treated animals

In our model, hypoxia/ischemia caused an approximately 2-fold increase in the mRNA expression of *Hif1a* and HIF1A-regulated *Bnip3.* DIM treatment (10 mg/kg) significantly decreased the expression of *Hif1a* and *Bnip3* to approximately 1-fold relative to the control level (Fig. 5a).

ELISA analysis revealed that neither hypoxia/ischemia nor DIM treatment changed the expression of HIF1A or CALPAIN-1 (Fig. 5b). Figure 5d presents the concentrations of selected proteins in pg/µl and fg/µl.

#### DIM decreases the protein concentration of HIF1A and CALPAIN-1 seven days after hypoxic/ischemic insult

ELISA analysis performed 7 days after hypoxia/ischemia revealed an increase in CALPAIN-1 protein concentration to 150% of control, whereas the concentration of HIF1A remained unchanged. The application of DIM (10 mg/kg) decreased the levels of HIF1A and CALPAIN-1 to 51% and 95% of the control levels, respectively (Fig. 5c, e).

### Effects of hypoxia/ischemia and DIM on the mRNA and protein expression levels of apoptotic and antioxidant factors three and seven days after the insult

#### DIM partially normalizes hypoxia/ischemia-induced changes in the mRNA and protein levels of pro-apoptotic and anti-oxidative factors three days after the insult

Hypoxia/ischemia-induced changes in the mRNA levels of selected genes were measured 3 days after insult. Hypoxia/ischemia decreased the mRNA expression of the anti-apoptotic gene *Bcl2* to 0.76-fold relative to the control level and increased the mRNA expression of the anti-oxidative enzyme *GPx3* to 2.3-fold relative to the control level. Hypoxia/ischemia did not evoke any changes in *Bax* or *Sod1* mRNA expression.

**Fig. 6:**
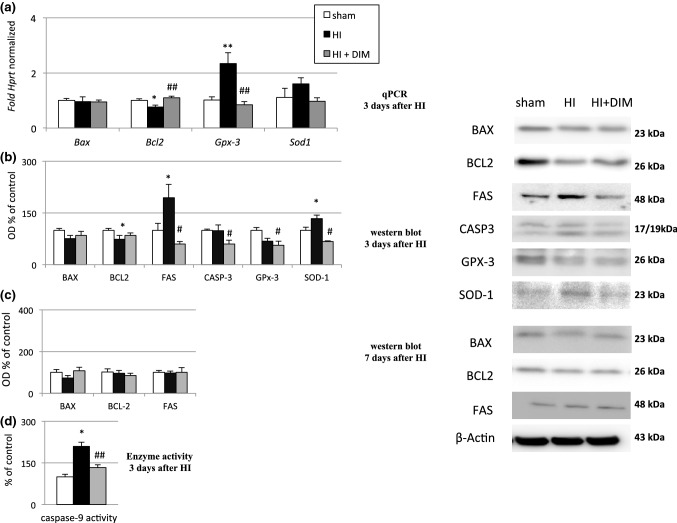
Effects of hypoxia/ischemia and DIM on the mRNA (**a**) and protein expression (**b–d**) of apoptotic and anti-oxidant factors three and seven days after insult. Seven-day-old rat pups were injected intraperitoneally (i.p.) with DIM 0.5 h, 24 h, 48 and 72 h after hypoxia/ischemia (HI) at a dose of 10 mg/kg of body weight. Sham-operated and HI control rats were injected with corn oil. Each bar represents the mean ± SEM. The number of replicates ranged from 2 to 3 (Western blotting) *p < 0.05, and **p < 0.01 versus sham animals, #p < 0.05 and ##p < 0.01 versus hypoxia/ischemia-treated animals

DIM (10 mg/kg) partially normalized the hypoxia/ischemia-induced changes, i.e., it increased the expression of *Bcl2* to 1-fold relative to the control level and inhibited the expression of *GPx3* to 0.8-fold relative to the control level. The mRNA expression of *Sod1* and *Bax* did not change after DIM treatment (Fig. 6a).

Using the western blot method, we demonstrated that hypoxia/ischemia increased the protein levels of FAS and SOD-1 to 194% and 133% of the control levels, respectively. Hypoxia/ischemia also induced a 30% decrease in BCL2 protein level. DIM partially inhibited these changes, i.e., it reduced the protein levels of FAS, CASP-3, GPx3 and SOD-1 to approximately 60% of the control levels (Fig. 6b). However it did not change protein levels of BAX and BCL2.

#### DIM does not evoke changes in the protein levels of BAX, BCL2 or FAS seven days after the insult

The protein levels of BAX, BCL2 and FAS measured 7 days after hypoxia/ischemia did not differ from the control values under untreated hypoxic/ischemic conditions or after DIM treatment (Fig. 6c).

#### DIM prevents hypoxia/ischemia-evoked caspase-9 activation

Three days after insult, hypoxia/ischemia increased caspase-9 activity to 210% of the control level. DIM (10 mg/kg) treatment decreased hypoxia/ischemia-evoked caspase-9 activity to 133% (Fig. 6d). The administration of DIM (10 mg/kg) to sham-operated animals did not result in any changes in caspase-9 activity (data not shown).

### DIM changes DNA methylation status in the rat brain undergoing hypoxia/ischemia

#### Under hypoxic/ischemic conditions, DIM causes the hypermethylation of global DNA

**Fig. 7:**
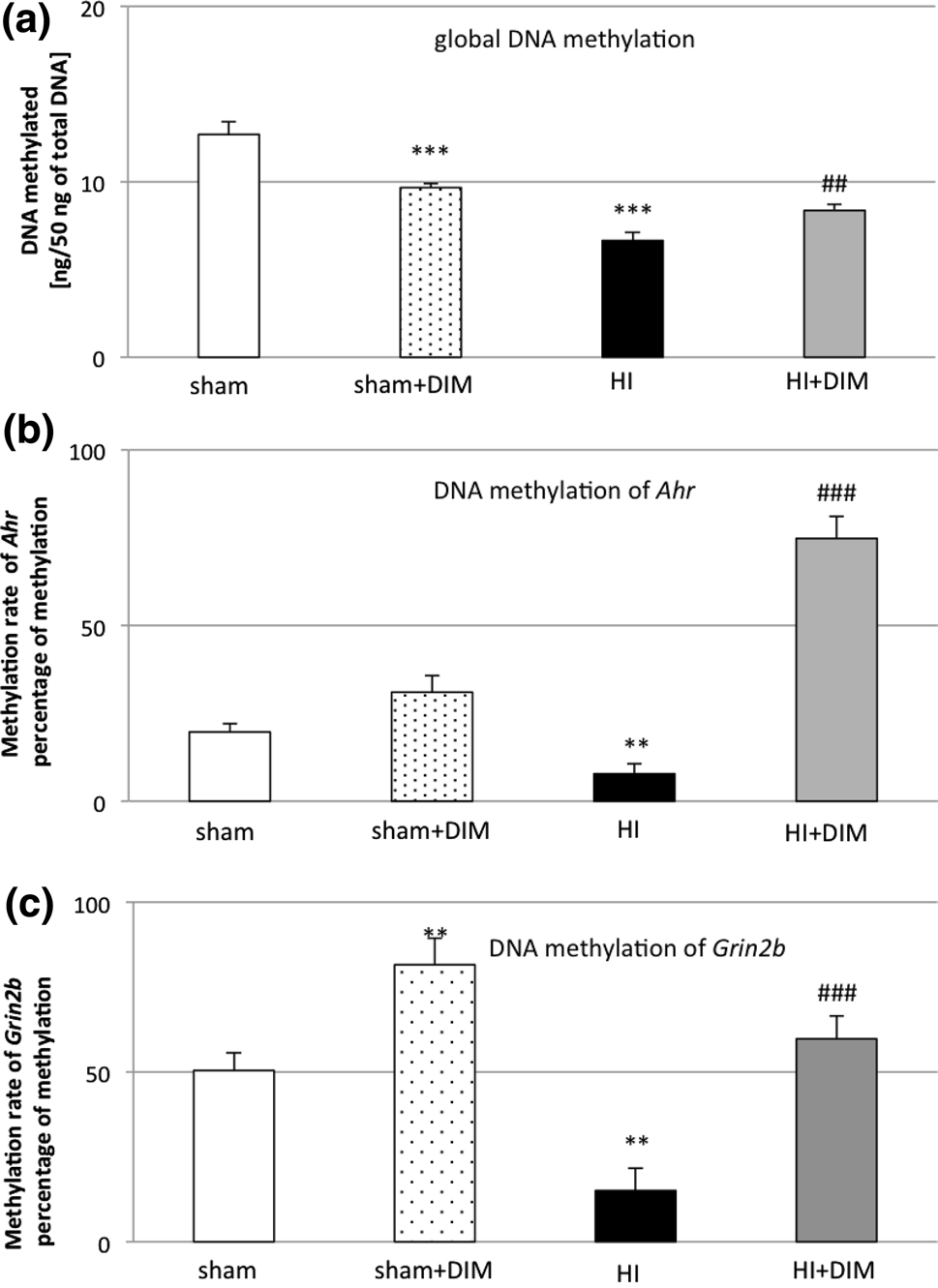
DIM and hypoxia/ischemia alter global DNA methylation (**a**) and the methylation rate of *Ahr* (**b**) and *Grin2b* (**c**) in the ipsilateral hemisphere. Seven-day-old rat pups were injected intraperitoneally (i.p.) with DIM 0.5 h, 24 h, 48 and 72 h after hypoxia/ischemia (HI) at a dose of 10 mg/kg of body weight. Sham-operated and HI control rats were injected with corn oil. Each bar represents the mean ± SEM. The number of replicates in each experiment was 6. **p < 0.01 and ***p < 0.001 versus sham animals ##p < 0.01, ###p < 0.001 versus hypoxia/ischemia-treated animals

In the sham-operated animals, global DNA methylation in the brain reached a value of 13 ng/50 ng purified DNA. After hypoxia/ischemia, global methylation was decreased to 6.6 ng. Treatment with DIM (10 mg/kg) increased DNA methylation to 8.4 ng. Interestingly, in sham-operated animals, DIM slightly reduced global methylation to 10 ng. (Fig. 7a).

#### Under hypoxic/ischemic conditions, DIM causes the hypermethylation of *Ahr* and *Grin2b* subunit of the NMDA receptor

In the subsequent experiments, we analyzed the methylation status of the DNA of selected genes in the rat brain undergoing hypoxia/ischemia. The levels of *Ahr and Grin2b* methylation in sham-operated animals was approximately 20% and 50%, respectively. Exposure of the rat brain to hypoxia/ischemia led to a decrease in *Ahr* and *Grin2b* methylation status to 7.8% and 15%, respectively. DIM treatment after hypoxia/ischemia evoked strong hypermethylation of the *Ahr and Grin2b* gene ranging from 60–75%. In the sham animals, DIM did not change the level of methylated *Ahr*, but it increased the level of methylated *Grin2b.* (Fig. 7b and c).

### DIM decreases the expression of *miR-181b* in the hypoxic/ischemic rat brain

**Fig. 8:**
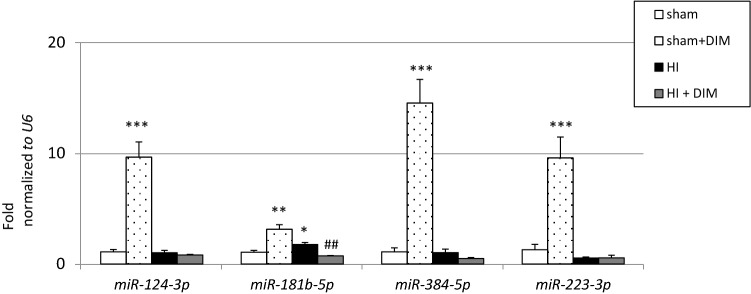
DIM and hypoxia/ischemia induce changes in miRNA expression levels in the rat brain. Seven-day-old rat pups were injected intraperitoneally (i.p.) with DIM 0.5 h, 24 h, 48 and 72 h after hypoxia/ischemia (HI) at a dose of 10 mg/kg of body weight. Sham-operated and HI control rats were injected with corn oil. Each bar represents the mean ± SEM. The number of replicates in each experiment was 4–6. *p < 0.05, **p < 0.01, and ***p < 0.001 versus sham animals ##p < 0.01 versus HI animals

In our model of perinatal asphyxia, hypoxia/ischemia increased *miR-181b* expression to 1.8-fold relative to the control level. However, it did not change the levels of *miR-124*, *miR-384*, or *miR-223*. DIM (10 mg/kg) treatment partially normalized hypoxia/ischemia-induced changes and decreased the expression of *miR-181b* to 0.7-fold relative to the control level. Interestingly, in the sham-operated animals, treatment with DIM significantly increased the expression of *miR-124*, *miR-181b*, *miR-384* and *miR-223* from 9-fold to 14-fold relative to the control level (Fig. 8).

### DIM does not change the protein levels of factors belonging to the AhR, NMDA and apoptosis signaling pathways in sham-operated animals

Western blot and ELISA analyses revealed that treatment with DIM did not evoke any changes in the protein expression of AhR, ARNT, HIF1A, CALPAIN-1, GluN1, GluN2B, BAX, BCL2, or CASP-3 in the sham-operated animals (Table 1).

**Table 1:** DIM does not change the protein levels of factors belonging to the AhR, NMDA and apoptosis signaling pathways in sham-operated animals

The level of proteins in sham animals % of control	AhR	ARNT	GluN1	GluN2B	HIF1A	CALPAIN-1	BAX	BCL2
Control	106 ± 6	100 ± 21	100 ± 1	100 ± 7	100 ± 15	100 ± 4	100 ± 8	100 ± 1
DIM treatment	90 ± 13	77 ± 35	89 ± 2	115 ± 12	103 ± 13	94 ± 6	99 ± 14	115 ± 2

Sham-operated rat pups were injected intraperitoneally (i.p.) with corn oil or DIM 0.5 h, 24 h, 48 and 72 h at a dose of 10 mg/kg of body weight. Each bar represents the mean ± SEM. The number of replicates in each experiment was 2–3 (Western blotting) and 6 (ELISA)

## Discussion

In this study, we demonstrated for the first time the strong neuroprotective capacity of DIM in an in vivo model of birth asphyxia, as evidenced by the restoration of the weight of the ipsilateral brain hemisphere, the normalization of the number of neurons in rat brains exposed to hypoxia/ischemia and the inhibition of mRNA expression of *Hif1a* and HIF1A-regulated *Bnip3* after postreatment with DIM. One may suggest that suppression of HIF1A signaling, which initiates apoptosis in severe hypoxia and interacts with AhR, is involved in neuroprotective effects of DIM in the ischemic brain [29, 30]. The rat model of perinatal asphyxia used in our study is clinically relevant because it corresponds to hypoxic/ischemic episodes in human newborns. In particular, the stage of development of 7-day-old rat pups corresponds to that of 32- to 36-week-old human fetuses [31]. Therefore, the neuroprotective effect of DIM posttreatment observed in our study in a rat model of perinatal asphyxia has strong translational value. It is particularly important because premature babies are most vulnerable to hypoxic/ischemic episodes and there is no effective treatment for hypoxia/ischemia in preterm and term newborns.

In the present study, perinatal asphyxia was accompanied by an increase in factors related to apoptosis and oxidative stress, which is in agreement with the results presented by other authors [32–34]. Our study demonstrated that DIM partially downregulated the expression of apoptotic factors, including FAS, CASP-3, and CAPN1, inhibited caspase-9 activity and increased the mRNA expression of anti-apoptotic *Bcl2*. All these data including the reduced expression of active form of caspase-3, which is a key apoptotic enzyme, strongly support anti-apoptotic property of DIM in the ischemic brain. There is no in vivo study with which our results can be compared. The only relevant data came from in vitro experiments which showed that DIM inhibited the expression of pro-apoptotic factors such as BAX, cytochrome c, cleaved caspase-3, phosphorylated form of p38 MAPK kinase, AIF and FAS in neurons subjected to hypoxia/ischemia or glutamate [6, 7, 35]. In addition to inhibition of apoptosis, DIM reduced the expression of anti-oxidant enzymes GPx3 and SOD-1. Paradoxically, newborns suffering from perinatal asphyxia express higher levels of anti-oxidative factors such as glutathione peroxidase, catalase, and superoxide dismutase. Nevertheless, these neonates experience higher degrees of oxidative stress, which leads to severe hypoxic-ischemic encephalopathy [12], and overexpression of SOD-1 in mouse brains increases perinatal asphyxia-induced damage [36]. Similarly, in our study, the rats subjected to perinatal asphyxia expressed higher levels of GPx3 and SOD-1 in the brain. We showed that DIM partially reversed these changes and inhibited the expression of these two enzymes, which we consider as positive and protective.

In the model of perinatal asphyxia used in our study, hypoxia/ischemia increased the expression of miR-181b, which is known to activate caspase-3-dependent apoptotic pathways and to inhibit the prosurvival PI3K/Akt signaling pathway [37]. This is in line with a report by O’Sullivan et al. [38], who showed upregulation of miR-181b in umbilical cord blood of newborns suffering from perinatal asphyxia. Therefore, targeting miR-181b could be a protective strategy against ischemic insult, as evidenced by the application of an antagomir [39, 40]. Our study demonstrated that DIM downregulated the expression of miR-181b, which could be one of the mechanisms of its neuroprotective action in perinatal asphyxia. Intriguingly, in nonischemic animals, DIM upregulated the expression of miR-223-3p involved in *Grin2b* degradation as well as increased methylation of *Grin2b* gene which links DIM to the control of excitotoxicity [41]. Moreover, in nonischemic rats, DIM stimulated expression of miRNAs such as miR-124, miR-384, and miR-181b, which are related to apoptosis, autophagy and/or excitotoxicity [42, 43]. Similarly, re-expression of miRNAs in response to DIM was demonstrated in cancer cell lines [44]. According to Glaich et al. [45], miRNAs encoded by highly methylated loci are more frequently expressed than miRNAs which are encoded by unmethylated loci. We suggest that in sham animals, DIM-evoked hypomethylation of global DNA could account for low expression of miRNAs, except for miRNAs, which control apoptosis, autophagy and/or excitotoxicity and inhibit the vulnerability of cerebral tissue to ischemic injury. In ischemic animals, DIM-evoked hypermethylation of global DNA could predispose brain tissue to synthesize large amounts of miRNAs, except for miR-181b-5p which was downregulated following the treatment with DIM.

In the present study, we show that the protective effects of DIM are accompanied by the inhibition of the AhR and NMDA signaling pathways, as indicated by reduced expression of *Ahr/*AhR, *Arnt/*ARNT, *Cyp1a1*/CYP1A1, GluN1 and *Grin2b*/GluN2B. This effect was correlated with enhanced global DNA methylation and, in particular, the increased methylation of the *Ahr* and *Grin2b* genes. *Ahr* and *Grin2b* hypermethylation suggest that DIM has property to silence the genes that could explain the role of DIM in controlling the AhR and NMDA signaling during hypoxia/ischemia. In addition, DIM has capacity to cause hypermethylation of *Grin2b* gene in normoxic/sham brain that supports neuroprotection against NMDA-mediated damage. Intriguingly, in our study, DIM also decreased expression of *Ahrr* mRNA encoding AHRR, which is known to repress AhR function and HIF-dependent signaling [46]. Because *Ahrr* gene was found to be silenced via hypermethylation [47], we suggest that in ischemic rats, DIM-evoked hypermethylation of global DNA could affect *Ahrr* gene to cause its downregulation. To our knowledge, there is no relevant *i*n vivo study to compare our data with. According to our previous results, DIM protects hippocampal neurons against hypoxia and ischemia *in vitro* through the inhibition of proteins in the AhR signaling pathway, including AhR, ARNT and CYP1A1 [6, 7] which is in line with our present in vivo results i.e., the downregulation of AhR signaling in DIM-treated rats subjected to birth asphyxia. DIM was found to cause widespread changes in promoter methylation patterns in prostate cells and to act as HDAC inhibitor in cancer cells [48, 49]. Previously, we showed that DIM has ability to reverse the ischemia-induced decrease in HDAC activity in mouse brain neurons [7]. Based on these data, one may assume that DIM changes the epigenetic status of ischemic brain not only via DNA hypermethylation as evidenced in the present study, but also by affecting histone acetylation.

There is no data on the involvement of NMDA signaling in the action of DIM during cerebral hypoxia/ischemia. The only data related to the findings of our study are associated with changes in the expression levels of NMDA receptors in a model of perinatal asphyxia. We showed that perinatal asphyxia increased the expression of GluN2B three and seven days after insult but did not change the expression of GluN1. Similarly, Guerguerian et al. [50] demonstrated that postnatal hypoxia/ischemia induced an increase in the GluN2B protein in the brains of newborn piglets. In contrast, Gurd et al. [51] observed a decrease in GluN2B expression in infant rats immediately after HI; however, the level of GluN1 remained unchanged, which is in accordance with our data. We suggest that the reduced expression of GluN2B observed by Gurd et al. [51] is related to the lack of reoxygenation or the more advanced stage of development of the rats compared to the rats used in our experiments.

In summary, this study demonstrated for the first time the neuroprotective capacity of 3,3’-diindolylmethane (DIM) in an *i*n vivo model of rat perinatal asphyxia, which has strong translational value and corresponds to hypoxic/ischemic episodes in human newborns. Posttreatment with DIM restored the weight of the ipsilateral hemisphere and normalized cell number in the rat brains exposed to perinatal asphyxia. The neuroprotective effect of DIM was mediated via inhibition of apoptosis and oxidative stress as well as downregulation of *Hif1a* mRNA (a hypoxic marker) and miR-181b (an indicator of perinatal asphyxia). Posttreatment with DIM also inhibited AhR and NMDA signaling, as indicated by the reduced protein levels and enhanced DNA methylation, both global and of specific genes. Because our study provided evidence that in rat brain undergoing perinatal asphyxia, DIM predominantly targets AhR and NMDA, we postulate that compounds that possess the ability to inhibit the signaling are promising therapeutic tools to prevent stroke.

## Abbreviations

AhR: Aryl hydrocarbon receptor
ARNT: Aryl hydrocarbon receptor nuclear translocator
CAPN1: Calpain-1
CASP-3: Caspase-3
DIM: 3,3′-Diindolylmethane
ELISA: Enzyme-linked immunosorbent assay
FAS: Fas cell surface death receptor
GPx3: Glutathione peroxidase 3
HI: Hypoxia/ischemia
HIF1A: Hypoxia-inducible factor 1 alpha
NMDA: *N*-methyl-D-aspartate receptor
SAhRM: Selective aryl hydrocarbon receptor modulator
SOD-1: Superoxide dismutase 1

## References

[1] Kurinczuk J, White-Koning M, Badawi N (2010). Epidemiology of neonatal encephalopathy and hypoxic-ischaemic encephalopathy. Early Hum Dev.

[2] Nair J, Kumar VHS (2018). Current and emerging therapies in the management of hypoxic ischemic encephalopathy in neonates. Children (Basel).

[3] Cuartero MI, Ballesteros I, de la Parra J, Harkin AL, Abautret-Daly A, Sherwin E, Fernández-Salguero P, Corbí AL, Lizasoain I, Moro MA (2014). L-kynurenine/aryl hydrocarbon receptor pathway mediates brain damage after experimental stroke. Circulation..

[4] Chen WC, Chang LH, Huang SS, Huang YJ, Chih CL, Kuo HC, Lee YH, Lee IH (2019). Aryl hydrocarbon receptor modulates stroke-induced astrogliosis and neurogenesis in the adult mouse brain. J Neuroinflammation.

[5] Anderton M, Manson M, Verschoyle R, Gescher A, Steward WP, Williams ML, Mager DE (2004). Physiological modeling of formulated and crystalline 3,3’-diindolylmethane pharmacokinetics following oral administration in mice. Drug Metab Dispos.

[6] Rzemieniec J, Litwa E, Wnuk A, Lason W, Krzeptowski W, Kajta M (2016). Selective aryl hydrocarbon receptor modulator 3,3’-diindolylmethane Impairs AhR and ARNT signaling and protects mouse neuronal cells against hypoxia. Mol Neurobiol.

[7] Rzemieniec J, Wnuk A, Lasoń W, Bilecki W, Kajta M (2019). The neuroprotective action of 3,3’-diindolylmethane against ischemia involves an inhibition of apoptosis and autophagy that depends on HDAC and AhR/CYP1A1 but not ERα/CYP19A1 signaling. Apoptosis.

[8] Wu QJ, Tymianski M (2018). Targeting NMDA receptors in stroke: new hope in neuroprotection. Mol Brain.

[9] Mueller-Burke D, Koehler RC, Martin LJ (2008). Rapid NMDA receptor phosphorylation and oxidative stress precede striatal neurodegeneration after hypoxic ischemia in newborn piglets and are attenuated with hypothermia. Int J Dev Neurosci.

[10] Johnston MV, Fatemi A, Wilson MA, Northington F (2011). Treatment advances in neonatal neuroprotection and neurointensive care. Lancet Neurol.

[11] Lin CH, Chen CC, Chou CM, Wang CY, Hung CC, Chen JY, Chang HW, Chen YC, Yeh GC, Lee YH (2009). Knockdown of the aryl hydrocarbon receptor attenuates excitotoxicity and enhances NMDA-induced BDNF expression in cortical neurons. J Neurochem.

[12] Kumar A, Ramakrishna SV, Basu S, Rao GR (2008). Oxidative stress in perinatal asphyxia. Pediatr Neurol.

[13] Eyileten C, Wicik Z, De Rosa S, Mirowska-Guzel D, Soplinska A, Indolfi C, Jastrzebska-Kurkowska I, Czlonkowska A, Postula M (2018). MicroRNAs as diagnostic and prognostic biomarkers in ischemic stroke-a comprehensive review and bioinformatic analysis. Cells.

[14] Godlewski J, Lenart J, Salinska E (2019). MicroRNA in brain pathology: neurodegeneration the other side of the brain cancer. Noncoding RNA.

[15] Rice JE, Vannucci RC, Brierley JB (1981). The influence of immaturity on hypoxic-ischemic brain damage in the rat. Ann Neurol.

[16] Bratek E, Ziembowicz A, Bronisz A, Salinska E (2018). The activation of group II metabotropic glutamate receptors protects neonatal rat brains from oxidative stress injury after hypoxia-ischemia. PLoS ONE.

[17] Wu TY, Huang Y, Zhang C (2015). Pharmacokinetics and pharmacodynamics of 3,3’-diindolylmethane (DIM) in regulating gene expression of phase II drug metabolizing enzymes. J Pharmacokinet Pharmacodyn.

[18] Leibelt DA, Hedstrom OR, Fischer KA, Pereira CB, Williams DE (2003). Evaluation of chronic dietary exposure to indole-3-carbinol and absorption-enhanced 3,3’-diindolylmethane in sprague-dawley rats. Toxicol Sci.

[19] Elackattu AP1, Feng L, Wang Z (2009). A controlled safety study of diindolylmethane in the immature rat model. Laryngoscope.

[20] Gamdzyk M, Ziembowicz A, Bratek E, Salinska E (2016). Combining hypobaric hypoxia or hyperbaric oxygen postconditioning with memantine reduces neuroprotection in 7-day-old rat hypoxia-ischemia. Pharmacol Rep.

[21] Kajta M, Wnuk A, Rzemieniec J, Lason W, Mackowiak M, Chwastek E, Staniszewska M, Nehring I, Wojtowicz AK (2019). Triclocarban disrupts the epigenetic status of neuronal cells and induces AHR/CAR-mediated apoptosis. Mol Neurobiol.

[22] Xie F, Xiao P, Chen D, Xu L, Zhang B (2012). miRDeepFinder: a miRNA analysis tool for deep sequencing of plant small RNAs. Plant Mol Biol.

[23] Wnuk A, Rzemieniec J, Staroń J, Litwa E, Lasoń W, Bojarski A, Kajta M (2019). Prenatal exposure to benzophenone-3 impairs autophagy, disrupts RXRs/PPARγ signaling, and alters epigenetic and post-translational statuses in brain neurons. Mol Neurobiol.

[24] Bratek E, Ziembowicz A, Salinska E (2018). Pretreatment with Group II metabotropic glutamate receptor agonist LY379268 protects neonatal rat brains from oxidative stress in an experimental model of birth asphyxia. Brain Sci.

[25] Rzemieniec J, Litwa E, Wnuk A, Lason W, Kajta M (2018). Bazedoxifene and raloxifene protect neocortical neurons undergoing hypoxia via targeting ERα and PPAR-γ. Mol Cell Endocrinol..

[26] Rzemieniec J, Litwa E, Wnuk A, Lason W, Gołas A, Krzeptowski W, Kajta M (2015). Neuroprotective action of raloxifene against hypoxia-induced damage in mouse hippocampal cells depends on ERα but not ERβ or GPR30 signalling. J Steroid Biochem Mol Biol.

[27] Wnuk A, Rzemieniec J, Lasoń W, Krzeptowski W, Kajta M (2018). Benzophenone-3 impairs autophagy, alters epigenetic status, and disrupts retinoid X receptor signaling in apoptotic neuronal cells. Mol Neurobiol.

[28] Eads CA, Danenberg KD, Kawakami K, Saltz LB, Blake C, Shibata D, Danenberg PV, Laird PW (2000). MethyLight: a high-throughput assay to measure DNA methylation. Nucleic Acids Res.

[29] Chen W, Ostrowski RP, Obenaus A, Zhang JH (2009). Prodeath or prosurvival: two facets of hypoxia inducible factor-1 in perinatal brain injury. Exp Neurol.

[30] Vorrink SU, Domann FE (2014). Regulatory crosstalk and interference between the xenobiotic and hypoxia sensing pathways at the AhR-ARNT-HIF1α signaling node. Chem Biol Interact.

[31] Patel SD, Pierce L, Ciardiello A, Hutton A, Paskewitz S, Aronowitz E, Voss HU, Moore H, Vannucci SJ (2015). Therapeutic hypothermia and hypoxia-ischemia in the term-equivalent neonatal rat: characterization of a translational preclinical model. Pediatr Res.

[32] Graham EM, Sheldon RA, Flock DL, Ferriero DM, Martin LJ, O’Riordan DP (2004). Neonatal mice lacking functional Fas death receptors are resistant to hypoxic-ischemic brain injury. Neurobiol Dis.

[33] Gamdzyk M, Makarewicz D, Słomka M, Ziembowicz A, Salinska E (2014). Hypobaric hypoxia postconditioning reduces brain damage and improves antioxidative defense in the model of birth asphyxia in 7-day-old rats. Neurochem Res.

[34] Bratek E, Ziembowicz A, Bronisz A, Salinska E (2018). The activation of group II metabotropic glutamate receptors protects neonatal rat brains from oxidative stress injury after hypoxia-ischemia. PLoS ONE..

[35] Lee BD, Yoo JM, Baek SY, Li FY, Sok DE, Kim MR (2019). 3,3’-Diindolylmethane promotes BDNF and antioxidant enzyme formation via TrkB/Akt pathway activation for neuroprotection against oxidative stress-induced apoptosis in hippocampal neuronal cells. Antioxidants (Basel).

[36] Ditelberg JS, Sheldon RA, Epstein CJ, Ferriero DM (1996). Brain injury after perinatal hypoxia-ischemia is exacerbated in copper/zinc superoxide dismutase transgenic mice. Pediatr Res.

[37] Liu J, Xing Y, Rong L (2018). miR-181 regulates cisplatin-resistant non-small cell lung cancer via downregulation of autophagy through the PTEN/PI3K/AKT pathway. Oncol Rep.

[38] O’Sullivan MP, Looney AM, Moloney GM, Finder M, Hallberg B, Clarke G, Boylan GB, Murray DM (2019). Validation of altered umbilical cord blood MicroRNA expression in neonatal hypoxic-ischemic encephalopathy. JAMA Neurol..

[39] Peng Z, Li J, Li Y, Yang X, Feng S, Han S, Li J (2013). Downregulation of miR-181b in mouse brain following ischemic stroke induces neuroprotection against ischemic injury through targeting heat shock protein A5 and ubiquitin carboxyl-terminal hydrolase isozyme L1. J Neurosci Res.

[40] Yuan L, Fan L, Li Q, Cui W, Wang X, Zhang Z (2019). Inhibition of miR-181b-5p protects cardiomyocytes against ischemia/reperfusion injury by targeting AKT3 and PI3KR3. J Cell Biochem.

[41] Harraz MM, Eacker SM, Wang X, Dawson TM, Dawson VL (2012). MicroRNA-223 is neuroprotective by targeting glutamate receptors. Proc Natl Acad Sci USA.

[42] Majdi A, Mahmoudi J, Sadigh-Eteghad S, Farhoudi M, Shotorbani SS (2016). The interplay of microRNAs and post-ischemic glutamate excitotoxicity: an emergent research field in stroke medicine. Neurol Sci.

[43] Fan J, Xu W, Nan S, Chang M, Zhang Y (2020). MicroRNA-384-5p promotes endothelial progenitor cell proliferation and angiogenesis in cerebral ischemic stroke through the delta-likeligand 4-mediated notch signaling pathway. Cerebrovasc Dis.

[44] Li Y, Sarkar FH (2016). MicroRNA targeted therapeutic approach for pancreatic cancer. Int J Biol Sci.

[45] Glaich O, Parikh S, Bell RE (2019). DNA methylation directs microRNA biogenesis in mammalian cells. Nat Commun.

[46] Vogel CFA, Haarmann-Stemmann T (2017). The aryl hydrocarbon receptor repressor - More than a simple feedback inhibitor of AhR signaling: clues for its role in inflammation and cancer. Curr Opin Toxicol.

[47] Zudaire E, Cuesta N, Murty V, Woodson K, Adams L, Gonzalez N, Martínez A, Narayan G, Kirsch I, Franklin W, Hirsch F, Birrer M, Cuttitta F (2008). The Aryl hydrocarbon receptor repressor is a putative tumor suppressor gene in multiple human cancers. J Clin Invest.

[48] Beaver LM, Yu T, Sokolowski E, Williams D, Dashwood R (2012). Ho E 3,3’-diindolylmethane, but not indole-3-carbinol, inhibits histone deacetylase activity in prostate cancer cells toxicol appl pharmacol. Toxicol Appl Pharmacol.

[49] Wong C, Hsu A, Buchanan A, Palomera-Sanchez Z, Beaver L, Houseman E, Williams D, Dashwood R, Ho E (2014). Effects of sulforaphane and 3,3’-diindolylmethane on genome-wide promoter methylation in normal prostate epithelial cells and prostate cancer cells. PLoS ONE.

[50] Guerguerian AM, Brambrink AM, Traystman RJ, Huganir RL, Martin LJ (2002). Altered expression and phosphorylation of N-methyl-D-aspartate receptors in piglet striatum after hypoxia-ischemia. Brain Res Mol Brain Res.

[51] Gurd JW, Bissoon N, Beesley PW, Nakazawa T, Yamamoto T, Vannucci SJ (2002). Differential effects of hypoxia-ischemia on subunit expression and tyrosine phosphorylation of the NMDA receptor in 7- and 21-day-old rats. J Neurochem.

